# The Sequential and Systemic Administration of BMP-2 and SDF-1α Nanocapsules for Promoting Osteoporotic Fracture Healing

**DOI:** 10.3390/biomimetics8040369

**Published:** 2023-08-16

**Authors:** Xiaolei Sun, Xueping Li, Peng Tian, Jin Zhao, Hou Xin, Xinlong Ma, Xubo Yuan

**Affiliations:** 1 Tianjin Key Laboratory of Composite and Functional Materials, School of Materials Science and Engineering, Tianjin University, Tianjin 300072, China; yangyabin@tiangong.edu.cn (X.S.); zhaojin@tju.edu.cn (J.Z.); houxin@tju.edu.cn (H.X.); 2 Institute of Biomedical Engineering, Chinese Academy of Medical Sciences and Peking Union Medical College, Tianjin 300192, China; lixueping@tju.edu.cn; 3 Department of Orthopaedics, Tianjin Hospital, Tianjin 300211, China; 0270012@fudan.edu.cn; 4 Tianjin Hospital, Tianjin University, No. 406 Jiefang South Road, Tianjin 300211, China

**Keywords:** osteoporotic fracture, BMP-2, SDF-1α, nanocapsules, systemic administration

## Abstract

Objective: The objective of this study was to investigate the use of the nanocapsule sequential delivery of BMP-2 and SDF-1α through the peripheral circulatory system to promote the healing of osteoporotic fractures. Methods: Based on increased vascular permeability in the early hematoma environment around the fracture and the presence of a large number of matrix metalloproteinase MMPs in the inflammatory environment, we designed MMP-sensitive nanocapsules which were formed viain situ free-radical polymerization on the surface of grow factors with 2-(methacryloyloxy) ethyl phosphorylcholine (MPC) and the bisacryloylated VPLGVRTK peptide. The antiphagic effect and biological activity of the growth factors for the nanomicrocapsule delivery system were tested by cell experiments. The 36 SD rats with an osteoporotic fracture model were randomly divided into six groups (A, B, C, D, E, and F). In this paper, the nanocapsules loaded with BMP-2 and SDF-1 are represented as n (BMP-2) and n (SDF-1α). In the six groups, the following different combinations of growth factors were injected into the bone defect site on days 1 and 3 after bone defect surgery: in group A, n (SDF-1α) combined with n (SDF-1α); in group B, n (BMP-2) combined with n (BMP-2); in group C, n (SDF-1α) + n (BMP-2) combined with n (SDF-1α) + n (BMP-2); in group D, n (SDF-1α) combined with n (BMP-2); in group E, n (BMP-2) combined with n (SDF-1α); in group F, nanocapsules without growth factor were used as the control group. Micro-CT was used to observe the effect of n(BMP-2) and n(SDF-1α) sequential delivery inearly healing in osteoporotic fractures. Finally, in this study, we evaluated the safety of the nanocapsules delivery system by detecting ectopic osteogenesis and inflammatory responses in animals. Results: Nanocapsules have low toxicity and protect the integrity and biological activity of growth factors. The results confirmed that nanocapsules could still be effectively targeted to the fracture site on days 1, 3, and 7 after intravenous administration. Growth factors encapsulated in nanocapsules have better bone repair results than natural growth factors. In particular, groups C and D had the best bone repair results than other groups.In vivo experiments confirmed that nanocapsules did not cause significant ectopic osteogenesis and inflammation. Conclusion: The results confirmed that the special vascular permeability and inflammatory factor microenvironment of the fracture site could be used to deliver two growth factors with a synergistic effect through venous circulation, which could better promote the healing process of osteoporotic fracture.

## 1. Introduction

As the global aging population increases, the medical expenses of osteoporotic fractureshave gradually become a heavy public health burden. It has been reported that growth factors such as BMP-2, IGFs, TGF-β, SDF-1α, and PDGF can promote bone healing in osteoporotic fractures [1]. In addition, previous studies have confirmed that the combined use of two growth factors could more effectively promote the bone repair process. Tan et al. proved that the local injection of SDF-1α enhanced bone healing induced by BMP-2 [2]. Wronski TJ et al. confirmed that sequential treatment with bFGF and PTH restored cancellous bone loss in severe osteoporosis [3]. In summary, the main delivery method of growth factors is local application at the fracture site, including local injection and sustained release materials. Using local administration is difficult when uniformly distributing growth factors into the hematoma formed at the fracture site, especially away from the injection site or the periphery of the fracture repair [4]. In addition, the local application of growth factors still has the following basic problems such as a short half-life, an uncontrolled release rate, inefficient accumulation at the fracture site, and infection [5]. More importantly, osteoporotic compression fractures are usually located deep, such as thoracolumbar compression fractures and hip fractures [6]. Therefore, the local application of growth factors is not suitable for complex osteoporotic fractures due to the lack of implantation space, thedifficulty in positioning, and the potential side effects at the fracture site.

The intravenous or subcutaneous injection of anti-osteoporosis drugs is usually used to promote the healing of osteoporotic fractures, mainly including bisphosphonates, estrogen, PTH, etc. [7]. However, the therapeutic process of anti-osteoporosis drugs is long, accompanied by a variety of serious side effects such as gastrointestinal reactions, mandibular necrosis, and atypical femoral fractures [8]. Except for anti-osteoporosis drugs, it has been reported that the systemic administration of FGF-1 (fibroblast growth factor-1) and BMP-7 (bone morphogenetic protein-7) promote the healing of osteoporotic fractures [9]. Relative to local delivery, the systemic administration of growth factors has the following advantages, including easy operability, precise dose control, repeated administration, and combined application. However, the systemic administration of growth factors has many limitations in promoting the healing of osteoporotic fractures. This is mainly due to the short half-life, adverse immune reactions, easy enzymatic hydrolysis, poor targeting and enrichment of the fracture site, and the ectopic osteogenesis of native growth factors during systemic administration [10,11]. A method that can protect growth factors with osteogenic activity from destruction by the body’s enzymes and immune system and yet effectively deliver the factors to a fracture site is urgently needed.

**Scheme 1:**
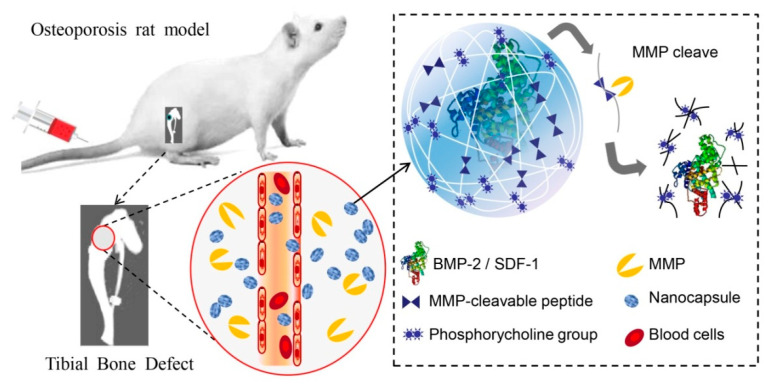
A schematic illustrating the release and degradation of MMP-responsive n (BMP-2) and n (SDF-1α) from blood vessels with increased permeability in the inflammatory environment (mainly containing MMP-2) of the bone defect site. The proximal tibial bone defect model was achieved in osteoporotic rats (ovariectomized, OVX).

To overcome the limitations of previous local delivery and systemic administration methods of native growth factors in promoting osteoporotic fracture healing, a systemic targeting delivery based on nanocapsule technology is a potential solution to improve treatment efficiency and reduce adverse effects. Actually, in the early stage of bone injury, the accumulation of inflammatory factors in the hematoma enhances the permeability of blood vessels at the fracture site [12]. At the same time, matrix metalloproteinases (MMPs) are largely secreted into the bone repair microenvironment to degrade proteins, while the content of MMPs in normal tissues and peripheral blood is low [13]. Based on the high permeability of blood vessels and overexpressed MMPs at the fracture site, we designed an injectable nanocapsule that was sensitive to MMPs and which could be administered intravenously and released through leaky blood vessels and accumulate within the fracture site [14]. As illustrated in Scheme 1, BMP-2 nanocapsules (denoted as n ((BMP-2)) or SDF-1α nanocapsules ((denoted as n (SDF-1α)) were synthesized viain situ polymerization with 2-(methacryloyloxy), ethyl phosphorylcholine (MPC), and a matrix metalloproteinase (MMP) cleavable peptide crosslinker.

However, the sequential and systemic delivery of two growth factors through the intravenous route to promote osteoporotic fracture repair has not been reported. In this study, as shown in Scheme 1, MMP-sensitive nanocapsules were used for the sequential and systemic delivery of BMP-2 and SDF-1α, which were then slowly released at the bone defect site to promote bone repair. The purpose of this study was to explore the synergistic effect of the systemic and sequential administration of two growth factors in promoting the healing of osteoporotic fractures, providing a new alternative treatment for difficult osteoporotic fractures in the clinic.

## 2. Materials and Methods

### 2.1. Materials

All chemicals used in this study were of an analytical reagent grade and purchased from Sigma-Aldrich (St. Louis, MO, USA) unless otherwise noted. BMP-2 and SDF-1α were purchased from Thermo Scientific (Carlsbad, CA, USA). Peptide was purchased from ChinaPeptides Co., Ltd. (Shanghai, China). TRIzol was supplied by Invitrogen, Inc. (Carlsbad, CA, USA). *N*-(3-aminopropyl)-methacrylamide was obtained from Polymer Science, Inc. (Crown Point, IN, USA). Phenyl-Sepharose. CL-4B was purchased from Solarbio Science & Technology Co., Ltd. (Beijing, China). An ALP ELISA kit was purchased from RayBiotech, Inc. (Norcross, GA, USA). The antibodies were supplied by Santa Cruz Biotechnology, Inc. (Santa Cruz, CA, USA).

### 2.2. Synthesis and Characteristics of n(BMP-2) and n(SDF-1α)

The MMP-sensitive nanocapsules were synthesized viain situ polymerization. Firstly, BMP-2 or SDF-1α sample was dissolved using 10 μL RNase-free water in an ice bath. During continuous stirring, the positive-charge monomer *N*-(3-Aminopropyl) methacrylamide (APM), 2-methacryloyloxyethyl phosphorylcholine (MPC), and crosslinker (bisacryloylated VPLGVRTK peptide) were sequentially added to the BMP-2 or SDF-1 (negatively charged) solution. Relying on the electrostatic interaction and hydrogenbonding interaction, these monomers and the crosslinker aggregated around BMP-2 or SDF-1α at 4 °C. Subsequent free-radical polymerization was initiated by adding 1 μL of 1% (*w*/*v*) ammonium persulfate (APS) and 2 μL of 1% (*w*/*v*) *N*,*N*,*N*′,*N*′-tetramethylethylenediamine (TEMED) to form the nanocapsules denoted as n (BMP-2) or n (SDF-1α). The final concentration of the BMP-2 or SDF-1α in RNase-free water was adjusted to 1 mg mL^−1^ in the solution. The synthesized nanocapsules were dialyzed against a 10-KDa molecular-weight dialysis bag in 4 °C PBS to remove unreacted monomers and the initiator. Agarose gel electrophoresis was used to detect whether the nanocapsules were effectively wrapped outside of BMP-2 or SDF-1α. Finally, images were collected with a 365 nm UV gel imaging system to observe the position of microRNA in the band. The size distribution and zeta potential of n (BMP-2) and n (SDF-1α) were measured using dynamic light scattering (DLS) (Brookhaven Instruments Ltd., Suffolk County, NY, USA), and samples were measured three times. The morphology of the samples stained with 2% phosphotungstic acid was assessed using a high-resolution transmission electron microscope (TEM, JEM-2100f, Jeol, Japan).

### 2.3. Biological Activity of BMP-2 and SDF-1α Released from Nanocapsules

The BMSCs (Bone Marrow Stromal Cells) at passage three were cultured in twelve-well plates at a density of 5 × 10^4^ cells per well for 2 days. The cells that reached approximately 70% confluence were incubated with BMP-2 n (BMP-2), and n (BMP-2) was treated with MMP. The final BMP-2 concentration of the medium was 100 μg mL^−1^. The BMSCs with no treatment were set as the control group. All groups were incubated with a DMEM medium containing 10% fetal bovine serum for 48 h. Then, each group of BMSCs was further incubated using an induction medium containing 100 nM dexamethasone, 50 μg mL^−1^ ascorbic acid, and 10 mM β-glycerophosphate. The induction medium was changed every three days. The BMSCs of all groups were examined by using ALP staining on day 14. The normalized intensity of ALP staining was determined by using ImageJ analysis software (version 1.53e). Finally, the quantitative analysis of the calcium deposition of the BMSCs was detected by using Alizarin Red staining using ImageJ on day 14 after induction. All samples were analyzed in triplicate.

The chemotaxis activity of encapsulated SDF-1α was measured with a Transwell with an 8 μm pore diameter. In total, 100 μL of BMSCs suspension were plated in the upper chambers of Transwell at a density of 1 × 10^5^/mL, and the lower chambers were supplemented with a serum-free low glucose- DMEM medium containing SDF-1α, n (SDF-1α) and n (SDF-1α) treated with MMP. The chamber containing only the DMEM medium was set as the control group. The 24-well plate was placed in a 5% carbon dioxide incubator and was incubated at 37 °C for 6 h. The Transwell chambers were washed with PBS and fixed with formaldehyde for 30 min. BMSCs were stained with a 0.1 % crystal violet solution for 20 min. Five fields were randomly observed using a 200× microscope, and the number of migrated BMSCs was counted in each group.

### 2.4. Cell Phagocytosis and Cytotoxicity of n (BSA)

To evaluate the phagocytic effect of inflammatory cells on nanocapsules, RAW 264.7 cells (1 × 10^4^ per well) were seeded into a 6-well plate containing coverslips and were cultured for an attachment at 37 °C. Nanocapsules encapsulating BSA (bovine serum albumin) instead of n (BMP-2) and n (SDF-1α) were used in the experiment. The nanocapsules encapsulating BSA were denoted as n (BSA). After 24 h, FITC-labeled native BSA and n (BSA) were added into the wells at a final concentration of 50 nM BSA for 4 h of incubation in an incubator. Subsequently, the cells were washed twice with PBS and stained with DAPI for 5 min. At last, the cells were fixed with 4% formaldehyde in PBS for 20 min at 4 °C and then washed with PBS three times. The coverslips were observed using Confocal Laser Scanning Microscopes-FV1000 (Olympus, Tokyo, Japan).

To evaluate the cytotoxic effect associated with the polymer shell, the cytotoxicity of nanocapsules in a series of concentrations was examined in BMSCs cells by using the MTT (3-(4, 5-Dimethylthiazol-2-yl)-2, 5-diphenyltetrazolium bromide) cell viability assay. To exclude any cytotoxic effects associated with growth factors, the nanocapsules encapsulating BSA (bovine serum albumin) replaced n (BMP-2) and n (SDF-1α). The nanocapsules encapsulating BSA were denoted as n (BSA). In total, 5 × 10^3^/well BMSCs were seeded in a 96-well plate and cultured in DMEM containing 10% FBS at 37 °C for 24 h. Subsequently, the DMEM medium in each group was replaced with the medium containing n (BSA) at final concentrations of 0 nM, 50 nM, 100 nM, and 200 nM. The wells without n (BSA) were used as the control group. Each group included six repetitions. After another 48 h, 20 μL of the sterile MTT solution at 5 mg/mL was added into each well, and the BMSCs were incubated for 4 h at 37 °C and 5% CO_2_. The unreacted MTT solution was removed and washed twice with PBS. In total, 150 μL of dimethyl sulfoxide (DMSO) was added to each well to dissolve the produced formazan crystals. The plate was placed in an incubator and shaken for 5 min on an orbital shaker. Finally, the optical density (OD) was obtained at 570 nm using a microplate reader (BioTek, Burlington, VT, USA). The OD of 6 wells in each group was measured. The cell activity was calculated through the following formula:

Cell viability (%) = (ODSample − ODBlank)/(ODControl − ODBlank) × 100%

### 2.5. Biodistribution and Bone Injury Targeting Ability of n (SDF-1α)

To study the accumulation efficiency of n (SDF-1α) at the fracture site, the tibial bone defect model was generated in ovariectomized (OVX) rats. Elderly female SD rats (14 months, 380 ± 10 g) were provided by the Experimental Animal Center of Tianjin University. The experimental protocol was approved by the Institutional Animal Care and Use Committee. All rats were anesthetized with an intraperitoneal injection of 6% sodium pentobarbital. After shaving and disinfecting the hind limb, the animals were subjected to the bilateral removal of their ovaries according to the classic osteoporosis model. Three months after surgery, the trabecular bone microarchitecture of the lumbar spine was measured using a microtomography CT (Inveon Siemens, Frankfurt, Germany) to evaluate the effect of the osteoporosis animal models. Under constant irrigation with 0.9 % saline, a round cortical and medullary defect with a diameter of 2 mm was created at the proximal tibia using a dental drill. After 24 h post-surgery, 9 rats were randomly divided into 3 groups. Two groups were, respectively, injected with 1 mL FITC-labeled SDF-1α and n (SDF-1α) (1 mg/kg) via the tail vein. The third group received the same amount of saline as the control. After 24 h of injection, the biodistribution and bone targeting ability of SDF-1α and n (SDF-1α) in vivo were observed using the IVIS imaging system. Finally, the animals were sacrificed, and the fluorescence intensity of the liver, kidney, and tibia of rats in each group was quantitatively detected using the IVIS imaging system. In the same methods as above, the biodistribution and accumulation efficiency of SDF-1α and n (SDF-1α) at the bone defect sites were detected. Subsequently, the accumulation efficiency of n (BMP-2) and n (SDF-1α) at the bone defect site was tested at 1, 3, and 7 days.

### 2.6. Bone Repair Efficiency of n (BMP-2) and n (SDF-1α) In Vivo

In total, 30 elderly SD rats (female, 14-month-old) were subjected to the bilateral removal of the ovaries (ovariectomized, OVX) according to the classic osteoporosis model. Five SD rats with only their skin cut and without the removal of their ovaries (shamoperation) served as a control group. Three months later, a high-resolution microcomputed tomography (micro-CT) system (Inveon Siemens, Germany) was used to evaluate whether the OVX model was successfully prepared.

Based on the OVX rat model, a bilateral tibial bone defects model was prepared according to the following method. After shaving and disinfecting the hind limb, a 5 mm incision through the skin was made on the proximal area of the tibia. A small surgical incision can avoid excessive damage to the soft tissue, thereby avoiding the targeting and aggregation of nanocapsules in the damaged soft tissue. Less damage to soft tissue can reduce the occurrence of ectopic osteogenesis. Under anesthesia (6% sodium pentobarbital, 60 mg/kg), all OVX rats were subjected to bilateral bone defects (2 mm diameter) in the proximal area of the tibia using a dental drill (SU 100, BEGO, Germany) under constant irrigation with 0.9% saline. After surgery, rats were individually caged and fed. All OVX rats with bilateral bone defects were randomly divided into 6 groups (groups A, B, C, D, E, and F), each with 6 rats. They were grouped as follows (Table 1).

To evaluate the efficacy of the sequential administration of BMP-2 and SDF-1α, n (BMP-2) (1 mg/kg body weight) and n (SDF-1α) (0.1 mg/kg body weight) were sequentially injected into the tail vein on the 1st and 3rd day after bone defects. In group A, the rats received an i.v. injection with n (SDF-1α) on days 1 and 3 after the bone defect. In group B, the rats received an i.v. injection with n (BMP-2) on days 1 and 3 after the bone defect. In group C, the rats received an i.v. injection with n (SDF-1α) + n (BMP-2) on days 1 and 3 after the bone defect, and the dosage of n (SDF-1α) and n (BMP-2) was both halved. In group D, the rats received an i.v. injection with n (SDF-1α) on the first day and then n (BMP-2) on the third day after the bone defect. In group E, the rats received an i.v. injection with n (BMP-2) on the first day and then with n (SDF-1α) on the third day after the bone defect. In group F, the rats received an i.v. injection with the same volume of nanocapsules without a growth factor (SDF-1α or BMP-2) as a control group.

After 4 weeks post-bone defect surgery, rats were euthanized to obtain the defect sites. The harvested samples were fixed in a 4% paraformaldehyde solution in PBS. A micro-CT system (Inveon Siemens, Germany) was used to scan the bone defect specimens with a section thickness of 21 μm and a voxel resolution of 22 μm^3^. The 3D reconstruction of the bone defect sites was created with standardized thresholds and morphological parameters. Three-dimensional images were re-digitized in a 16-bit data format and visualized. The newly formed bone tissue within 2 mm of diameter at the bone defect was defined as the region of interest (ROI). Bone volume/total volume (BV/TV; %) and trabecular thickness (Tb.Th; μm) were measured using the analysis software micro CT.

### 2.7. Inflammation and Ectopic Osteogenesis of n (BMP-2) and n (SDF-1α) In Vivo

In this study, X-ray whole-body imaging and the HE staining of muscle tissue around bone defects were used to observe ectopic osteogenesis after tail vein injection in the rats in each experimental group. At the same time, we observed the inflammatory factors in the peripheral blood of rats 24 h after tail vein injection. The vital signs and survival status of the rats in each group were carefully monitored. In the fourth week after tail vein injection, the ectopic osteogenesis of the rats in each group (A, B, C, D, E, and F group) was detected by using an X-ray. Subsequently, HE staining was used to observe the morphology of the muscle around the bone defect site. After 24 h post-tail vein injection, peripheral blood was collected via the orbital venous plexus, and serum was collected via centrifugation. The inflammatory mediators IL-6 and TNF-α in the serum of each group were quantitatively determined using an ELISA kit. Using the same methods as above, the effects of different concentrations of SDF-1αand n (SDF-1α) on inflammatory response in rats were determined.

### 2.8. Statistics

All the experiments were repeated 3 times independently. Additionally, all data are presented as the mean ± standard deviation (SD). Data were compared by using a two-tailed *t*-test or one-way ANOVA for experiments with more than two subgroups. The SPSS 22.0 statistical software (IBM Corp. Released 2013. IBM SPSS Statistics for Windows, Version 22.0. Armonk, NY, USA: IBM Corp.) was developed for data analysis. Differences with a value of *p* < 0.05 were considered significant.

## 3. Results

### 3.1. Characteristics of n (BMP-2) and n (SDF-1α)

**Figure 1:**
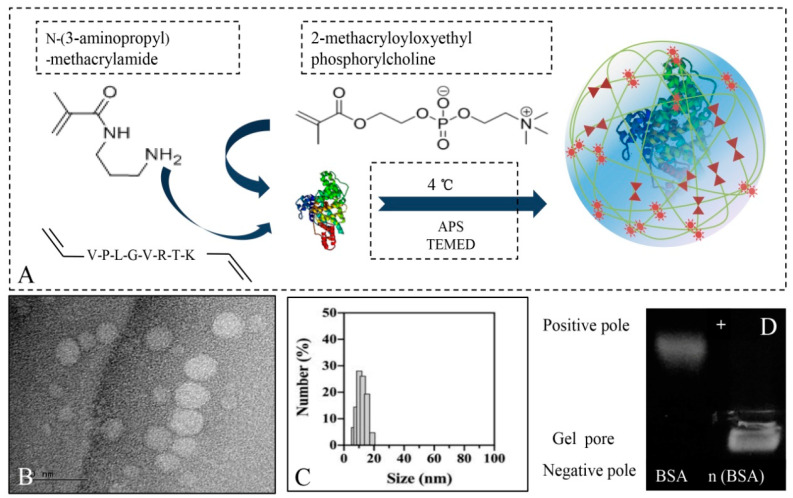
(**A**) A schematic illustrating the synthesis mechanism of MMP-responsive to n (BMP-2) and n (SDF-1α). (**B**) A representative TEM image of n (BMP-2) (scale bar, 50 nm). (**C**,**D**) Zeta potential and agarose gel electrophoresis of n (BMP-2) (molarratio ¼ 4000:1).

The preparation process of n (BMP-2) and n (SDF-1α) is shown in Figure 1A. In order to observe the characteristics of nanocapsules consistently, BSA was used instead of BMP-2 and SDF-1α to synthesize nanocapsules and test their characteristics. The nanocapsules loaded with BSA are represented as n (BSA). Using APM and MPC as monomers and the bisacrylated MMP-2-responsive peptide as the crosslinking, the n (BSA) was synthesized by using in situ polymerization. Under an electrostatic interaction and hydrogenbonding interaction, the monomer and crosslinker formed a degradable polymer shell around the surface of the BSA at 4 °C. APS and TEMED initiated an in situ polymerization reaction between the monomers and crosslinker to form n (BSA).

The transmission electron microscope (TEM) observed that the n (BSA)were regular spherical nanocapsules with a uniform diameter of 10~20 nm and no adhesion (molar ratio = 4000:1) (Figure 1B). The dynamic light scattering (DLS; ∼12.5 nm) measurement was consistent with the TEM observation (Figure 1C). As an acidic protein, the BSA is electronegative in a physiological state. The gel electrophoresis assay showed that native BSA shifted to thepositive electrode, while the n (BSA) (prepared with the M:R ratios 4000:1) were trapped in the loading wells without a significant electrophoresis shift (Figure 1D). The reason for this is that the zeta potential of n (BSA) was close to neutral due to the shielding effect of the MPC-based polymer shell. Therefore, the above results confirm that the nanocapsules have a uniform spherical structure with appropriate size and zeta potential. The nanocapsules had satisfactory encapsulation effects on BSA (including BMP-2 and SDF-1α).

### 3.2. Quantitative Determination of Biological Activity of BMP-2 and SDF-1α Released from Nanocapsules

**Figure 2:**
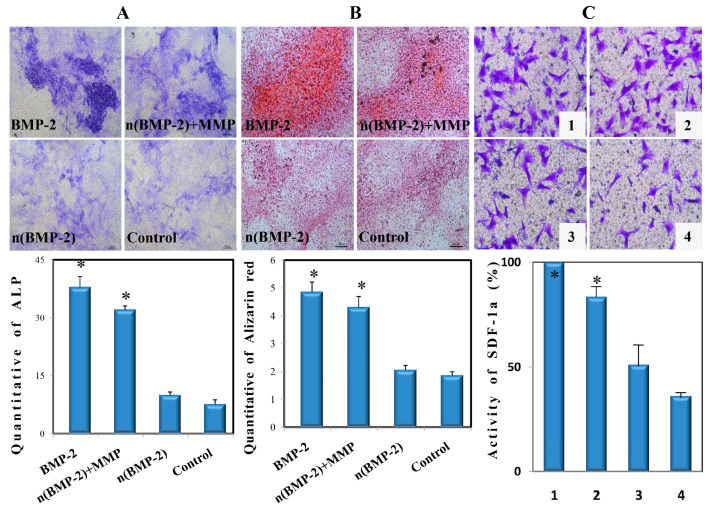
(**A**) ALP staining was performed in BMSCs of BMP-2, n (BMP-2) with MMP, n (BMP-2) and the control group (40×), where the quantitative analysis of ALP staining was performed using ImageJ (* *p* < 0.05 vs. n (BMP-2) and control group). (**B**) Alizarin red staining analysis was performed in BMP-2, n (BMP-2) with MMP, n (BMP-2), and the control group (40×), and the quantitative analysis of alizarin red staining was performed using ImageJ (* *p* < 0.05 vs. n (BMP-2) and control group). (**C**) Migration experiments of BMSCs in SDF-1α, n (SDF-1α) with MMP, n (SDF-1α), and the control group (40×), and the quantitative analysis of the migration experiments was determined using ImageJ (* *p* < 0.05 vs. control group).

BMSCs were incubated with BMP-2 and n (BMP-2), and n (BMP-2) was pretreated with MMP at the same culture medium for 48 h. A blank control group without BMP-2 was established. After 48 h of culture, ALP staining and normalized intensity using ImageJ software demonstrated that the activity of BMP-2 of n (BMP-2) when pretreated with the MMP group was significantly higher than n (BMP-2) and the blank control group (Figure 2A). At the same time, alizarin red staining was quantitatively analyzed using ImageJ software to evaluate the activity of BMP-2 releases from the nanocapsules. The results showed that the calcium deposition of BMSCs significantly increased in n (BMP-2) when pretreated with the MMP group (Figure 2B). The above results indicate that the degraded nanocapsules could release bioactive BMP-2.

The cell chemotaxis experiment uses co-cultured Transwell chambers (including the upper chamber and the lower chamber, with a pore diameter of 8 μm) to observe the migration rate of cells. Cells migrate toward the side with the high biological activity of growth factors. The higher the cell migration rate, the better the biological activity of growth factors. The SDF-1α-free medium was added to the lower chamber of Transwell, and after 6 h of incubation, almost no BMSCs migrated from the upper chamber to the lower chamber (Figure 2C). The results show that 100 ng/mL SDF-1α had the strongest chemotaxis on BMSCs, and the chemotaxis was set at 100%. Compared with the 100 ng/mL SDF-1α group, the chemotaxis of n (SDF-1α) treated with the MMP group was 82%. The chemotaxis of the n (SDF-1α) and blank control groups were 28% and close to 0%. The results showed that the nanocapsule structure could be degraded by MMP and release SDF-1α with high activity.

### 3.3. The Results of Cell Phagocytosis and Cytotoxicity of n (BSA) In Vitro

**Figure 3:**
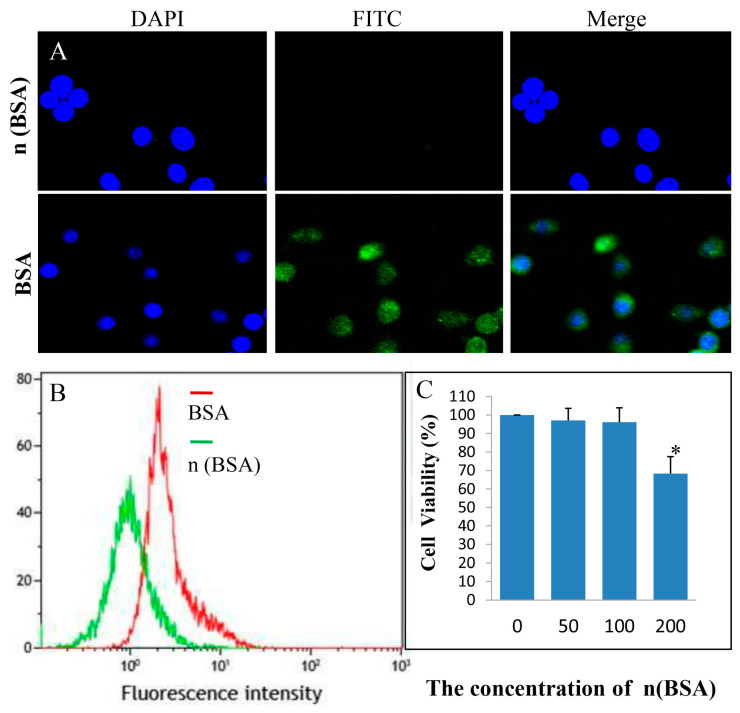
(**A**) Intracellular uptake analysis of the nanocapsules as assessed by using confocal microscopy after 24 h of incubation with 50 Nm FAM (carboxy fluorescein)-labeled (scale bar, 200 μm). (**B**) The results of phagocyte experiments show that nanocapsules could significantly reduce the phagocytosis of BSA by macrophages. (**C**) The cytotoxicity of n (BSA) in BMSCs with different concentrations of n (BSA) (* *p* < 0.05 vs. the concentrations of 50, 100, and 200 nM).

In this study, the protective efficiency of nanocapsules on growth factors was observed by using a RAW 264.7 cell phagocytosis experiment. To simplify this part of the experiment, BSA (Bovine serum albumin) was used instead of n BMP-2 and SDF-1α to serve as the encapsulation factor for nanocapsules. The fluorescein-labeled BSA (FITC-labeled BSA and FITC-labeled n (BSA)) was used to evaluate the phagocytic effect of macrophages on nanocapsules. The results of the phagocyte experiments that used RAW 264.7 showed that natural BSA was greatly engulfed into the cytoplasm by the macrophages. On the contrary, only a small amount of n (BSA) was engulfed by macrophages in the cytoplasm (Figure 3A). The results of the phagocyte experiments showed that nanocapsules could significantly reduce the phagocytosis of BSA by macrophages. At the same time, flow cytometry results also showed that there was a significant difference in phagocytosis between natural BSA and n (BSA) (Figure 3B). The MTT assay showed that the cell proliferation rate of each group was basically similar to that of the control group. This result confirmed that different concentrations of nanocapsules do not cause toxicity to cell proliferation (Figure 3C).

### 3.4. The Accumulation Efficiency and Bone Targeting Ability of n (SDF-1α)

**Figure 4:**
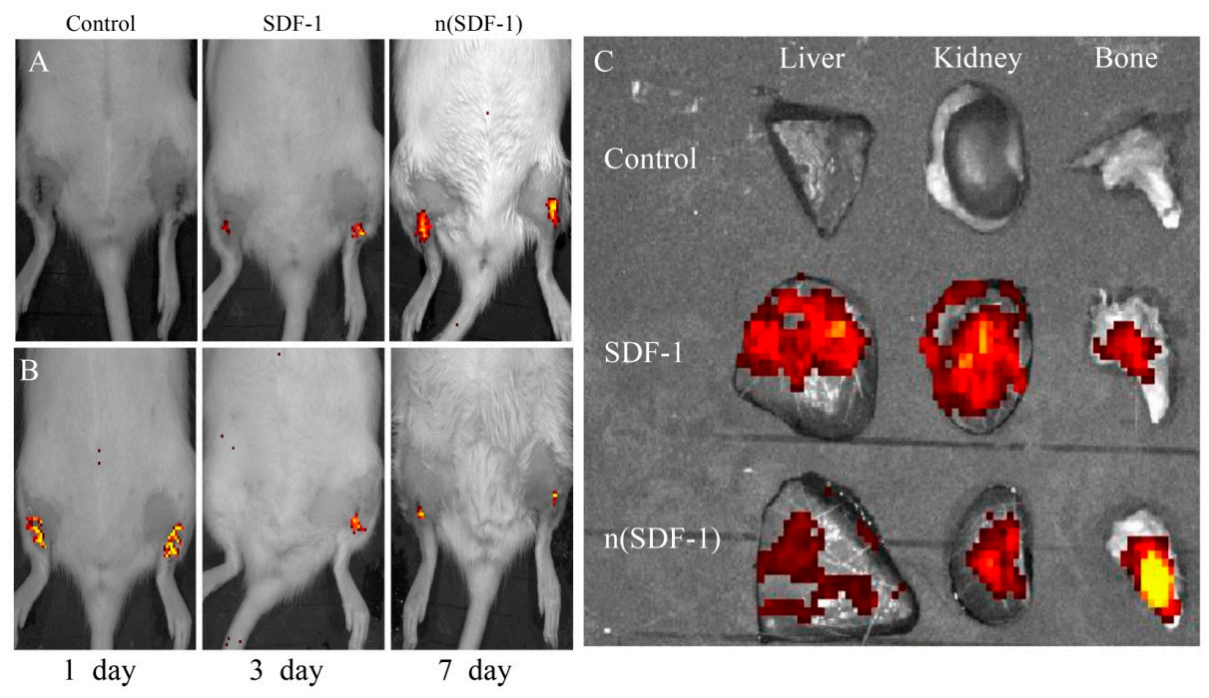
(**A**) Fluorescence intensity of bone defect in rats in SDF-1α, n (SDF-1α), and the control group at 24 h after injection through the tail vein. (**B**) Fluorescence intensity of bone defect of FITC-labeled n (SDF-1α) on 1, 3, and 7 days after tail vein injection. (**C**) Fluorescence intensity of SDF-1α, n (SDF-1α), and the control group in liver, kidney, and bone tissues.

In this study, we observed the accumulation efficiency of n (SDF-1α) at the site of bone defects in rats. First, we observed the accumulation efficiency of FITC-labeled SDF-1α, FITC-labeled n (SDF-1α), and the control group (saline) at the site of bone defects in rats after tail vein injection for 24 h. At the same time, we observed the duration of FITC-labeled n (SDF-1α) at bone defects on days 1, 3, and 7 after tail vein injection. After 24 h post-injection through the tail vein, the results showed that SDF-1α could accumulate in small amounts at the bone defect site. Due to the protection of nanocapsules, the accumulation efficiency of n (SDF-1α) could be significantly increased at the bone defect site. The control group (injected with physiological saline) did not exhibit aggregation (Figure 4A). Meanwhile, we also tested the duration of FITC-labeled n (SDF-1α) in bone defect sites on days 1, 3, and 7. The results showed that n (SDF-1α) still aggregated at the bone defect site on the 7th day after tail vein injection (Figure 4B). The results show that n (SDF-1α) clustered significantly less in the liver and kidney than in the native SDF-1α. The results show that n (SDF-1α) accumulated significantly higher in the bone defect site than in the native SDF-1α group and control group (Figure 4C). In summary, nanocapsules can effectively protect the growth factors transported by the peripheral blood system and achieve accumulation efficiency at the fracture site.

### 3.5. The Results of Bone Repair Efficiency of n (BMP-2) and n (SDF-1α) In Vivo

**Figure 5:**
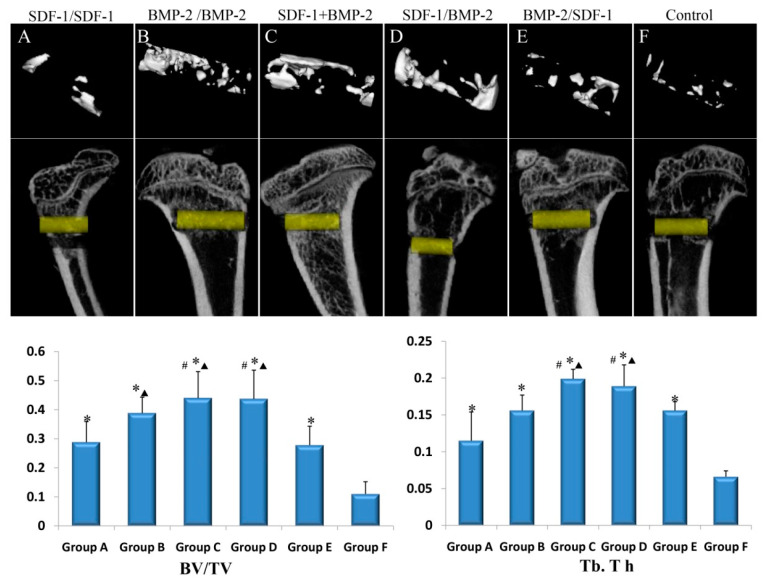
Micro-CT images of representative rat tibias in the 4th week: the 3D architecture of trabecular within the distal tibias in (**A**–**F**). Quantitative analysis of the new trabecular bone volume (BV/TV) and trabecular thickness (Tb.Th) of the bone defect in OVX rats by using microtomography analysis (* *p* < 0.05 vs. control group; ^▲^ *p* < 0.05 vs. E group; **^#^** *p* < 0.05 vs. A group).

Four weeks after the tail vein sequential injection of n (BMP-2) and/or n (SDF-1α), micro-CT results showed that different amounts of newborn trabeculae were generated at the tibial bone defect in all groups (including the control group). A quantitative analysis of a microscopic CT 3D reconstruction showed higher BV/TV and Tb.Th in five experimental groups (A, B, C, D, and E group) than the control group (Figure 5). Particularly, BV/TV was higher in groups C and D than in groups A and E. Meanwhile, Tb.Th in groups C and D was higher than in group A.

### 3.6. The Test of Inflammation and Ectopic Osteogenesis In Vivo

**Figure 6:**
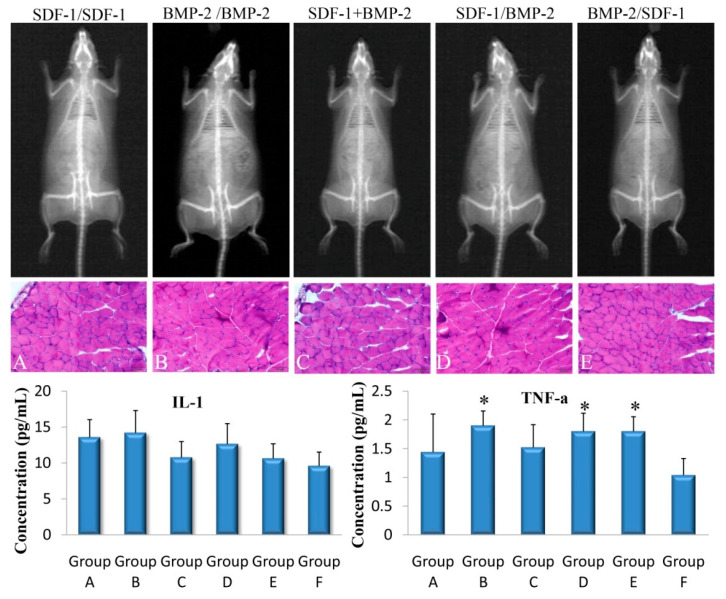
X-ray results confirmed that ectopic osteogenesis was not observed in all experimental groups after 4 weeks of injection through the tail vein. HE staining of muscle tissue also showed no ectopic ossification in each group (the scale bar was 200 μm). The ELISA results showed no statistical difference in IL-6 levels in each group compared with the control group; ELISA results showed a statistical difference in TNF- α in groups B, D, and E compared with the control group (* *p* < 0.05 vs. control group).

In this study, X-ray whole-body imaging and the HE staining of muscle tissue around bone defects wereused to observe ectopic osteogenesis after tail vein injection in rats for each experimental group. At the same time, we observed the inflammatory factors in the peripheral blood of rats 24 h after tail vein injection. The X-ray scanning results confirmed that no ectopic osteogenesis occurred in all experimental groups. The results of the HE staining of muscle tissue in each group did not show a positive calcification in muscular or intermuscular tissue. Finally, the ELISA results showed that there was no statistically significant difference in the IL-6 level in all experimental groups. Meanwhile, ELISA results showed a statistical difference in TNF- α for groups B, D, and E compared with the control group (Figure 6).

## 4. Discussion

Unlike the healing process of young patients, elderly patients with osteoporotic fractures have poor healing ability, low stem cell mobilization ability, and poor nutritional status, resulting in an unsatisfactory microstructure in the newly regenerated bone [15]. Therefore, in the elderly, the above degenerative repair ability has caused terrible clinical problems such as delayed healing, nonunion, and even high disability and mortality [16]. BMP-2 has been shown to have a strong ability to promote bone repair in vivo [17]. In addition, SDF-1α was known to have the strongest ability to promote vascularization and also had the effect of promoting osteogenesis [18]. However, BMP-2 is known to be administered in high, non-physiological doses in order to achieve adequate bone repair in research or clinical applications, which could lead to significant side effects [19]. At the same time, the bone repair microenvironment of elderly patients with osteoporosis is worse than that of the normal bone repair microenvironment, and the structure and strength of repaired bone tissue are also worse. Based on the disadvantages of the local delivery of BMP-2 and SDF-1α, it is feasible to reduce the dose of BMP-2 required to promote bone formation. In this study, BMP-2 and SDF-1α were delivered by the peripheral circulatory system to promote vascularization and bone repair through the sequential application of two growth factors with synergistic effects. We explored the systemic sequential delivery of two growth factors to promote osteoporotic fractures that were more difficult to heal.

This study is based on the fact that the amino groups that are present at both ends of the peptides are able to promote their bisacryloydation. At the same time, the middle fraction of both peptides is sensitive to MMP. The results showed that there was a minimal effect relationship between the envelope of growth factors and the dose of MPCs. However, when the molar ratio was higher, most of the BMP-2 was not released from the nanocapsule. A possible reason for this result is that the large number of MPCs effectively inhibited the adsorption of enzymes on the nanocapsules. In this study, the same dose of BMP-2, n (BMP-2), and n (BMP-2) with MMP was used to culture mesenchymal stem cells, respectively. The results of ALP staining on mesenchymal stem cells showed that the osteogenesis effect of the n (BMP-2) group without MMP and the blank control group was poor. This also confirmed that BMP-2 in the nanocapsule was fully released into the cell culture medium under the action of MMP. At the same time, similar results were shown in cell migration experiments in co-cultured Transwell chambers. Cell experiments confirmed that MMP could unpack the nanocapsule shell to fully release SDF-1α, and this growth factor maintains good biological activity. In previous studies, the delivery of bone repair growth factors through peripheral circulation has not been well applied. The main reasons for this were inefficient delivery and potential systemic side effects [20,21].

The results of macrophage phagocytosis experiments showed that nanocapsules can effectively reduce the phagocytosis of macrophages. The possible reason for this is that nanocapsules can effectively reduce the adsorption of growth factors on proteins. By effectively isolating the adsorption of growth factors by nanocapsule polymer shells, it can help growth factors avoid being engulfed by immune cells or macrophages in the peripheral circulation [22]. However, studies have confirmed that even with the protection of the polymer shell, growth factors can still be partially adsorbed by proteins or bound by immune cells. In addition, the results of culturing RAW 264.7 cells with different concentrations of n (BSA) showed that the cell proliferation rate of each concentration group was basically similar to that of the control group. The damage to the tissues surrounding the fracture caused the destruction of a large number of arteriovenous vessels and capillaries. There were a large number of inflammatory factors brought about by the peripheral circulation penetration or local inflammatory reactions in the hematoma microenvironment around the fracture [23,24]. Hu et al. proved that a variety of inflammatory mediators at the site of bone injury, such as interleukin, significantly increased the permeability of the vascular barrier, resulting in macromolecules and even cells in blood vessels being more conducive to migrating to the injured part [25]. Using this special hematoma microenvironment and a highly permeable vascular wall, multiple administrations of multiple growth factors could be achieved via passive delivery. Our results also confirmed that nanocapsules can significantly improve the ability of growth factors to target bone damage. However, we also found that BMP-2 and SDF-1α have the ability to passively target fracture sites. Nanocapsule delivery growth factors can penetrate more and become more enriched at the site of injury than native growth factors.

The results of this experiment confirmed that n (BMP-2) still had a good target enrichment effect at the bone injury site on the 1st, 3rd, and 7th days after fracture. It has been shown that the early stage of bone injury is a critical period for the initiation of the fracture repair process, and both BMP-2 and SDF-1α play a key role in the early initiation of fracture healing [26]. Therefore, the delivery of two synergistic growth factors through the peripheral circulation early in the fracture can promote the process of early fracture healing. In addition, our results also found that the targeted enrichment efficiency of n (BMP-2) and n (SDF-1α) at the bone injury site gradually decreased with the extension of bone injury time. Possible causes are related to vascular repair at the site of injury after fracture and vascular permeability repair due to hematoma organization. The results of this study confirm that the nanocapsule encapsulated delivery of growth factors can better evade immune clearance in vivo. Both n (BMP-2) and n (SDF-1α) can be more targeted and rarely aggregate the fracture site within the liver and kidney.

Recombinant human BMP-2 and BMP-7 were approved by the FDA for the treatment of spinal fusion and long bone nonunion [27]. Unfortunately, BMP-2 is typically delivered in supraphysiological doses (as high as 12 mg per collagen scaffold) [28] and can result in significant adverse side effects [29]. In order to reduce the dosage of BMP-2, it is a feasible strategy to use another growth factor together. Stromal cell-derived factor-1 (SDF-1α) is up-regulated after fracture and is important for stem cell homing at the fracture site through CXC chemokine receptor type 4 (CXCR4) [30].

The results of this study confirm that the sequential application of two growth factors can promote the healing of osteoporotic fractures in rat models. Many studies have confirmed that the local combined application of BMP and SDF-1α can effectively promote bone repair [31]. However, the complex fracture repair environment in the human body requires that the nanocapsules delivery system has a longer cycle time and the best effective dosing regimens. In particular, the application time interval and maximum dose of the two factors need to be further explored in clinical applications.

The results confirmed that no ectopic osteogenesis occurred in any group of animal models. Compared with the local delivery of high concentrations of growth factors, the dose and concentration of peripheral circulation are small and do not lead to ectopic osteogenesis. Another possible reason is that the peripheral circulation delivery time is longer, and the nanocapsule has a slow-release effect. The biosafety of the nanocapsule delivery of growth factors to promote bone repair growth factors can promote the healing of osteoporotic fractures, which was further confirmed via X-ray, muscle tissue HE staining, and the detection of inflammatory factors.

## 5. Conclusions

The systemic delivery of MMP-sensitive n (BMP-2) and n (SDF-1α) could effectively target growth factors to bone defect sites. The structure and functionality of BMP-2 and SDF-1α were well maintained during systemic delivery. These results confirmed that the simultaneous delivery of n (SDF-1α) and n (BMP-2) and the delivery of n (SDF-1α) and then n (BMP-2) had a better effect inpromoting bone repair than other groups. The systemic delivery nanocapsules can be widely used in non-surgical elderly patients with osteoporotic fractures.

## Figures and Tables

**Table 1:** Explanation of animal experiment grouping.

Groups (*n* = 6)	24h after Bone Defects	3rd Day after Bone Defects
Group A	n (SDF-1α)	n (SDF-1α)
Group B	n (BMP-2)	n (BMP-2)
Group C	n (SDF-1α) + n (BMP-2)	n (SDF-1α) + n (BMP-2)
Group D	n (SDF-1α)	n (BMP-2)
Group E	n (BMP-2)	n (SDF-1α)
Group F	Nanocapsules without growth factor	Nanocapsules without growth factor

## Data Availability

The data that support the findings in this study are available from the corresponding authors upon reasonable request.
